# Prolonged Interruption of Cognitive Control of Conflict Processing Over Human Faces by Task-Irrelevant Emotion Expression

**DOI:** 10.3389/fpsyg.2017.01024

**Published:** 2017-06-20

**Authors:** Jinyoung Kim, Min-Suk Kang, Yang Seok Cho, Sang-Hun Lee

**Affiliations:** ^1^Cognitive and Systems Neuroscience Lab, Department of Brain and Cognitive Sciences, Seoul National UniversitySeoul, South Korea; ^2^Department of Psychology, Sungkyunkwan UniversitySeoul, South Korea; ^3^Center for Neuroscience Imaging Research, Institute for Basic ScienceSuwon, South Korea; ^4^Department of Psychology, Korea UniversitySeoul, South Korea

**Keywords:** emotion expression, cognitive control, emotional saliency, emotion regulation, emotional conflict, selective attention, human face, congruency effect

## Abstract

As documented by Darwin 150 years ago, emotion expressed in human faces readily draws our attention and promotes sympathetic emotional reactions. How do such reactions to the expression of emotion affect our goal-directed actions? Despite the substantial advance made in the neural mechanisms of both cognitive control and emotional processing, it is not yet known well how these two systems interact. Here, we studied how emotion expressed in human faces influences cognitive control of conflict processing, spatial selective attention and inhibitory control in particular, using the Eriksen flanker paradigm. In this task, participants viewed displays of a central target face flanked by peripheral faces and were asked to judge the gender of the target face; task-irrelevant emotion expressions were embedded in the target face, the flanking faces, or both. We also monitored how emotion expression affects gender judgment performance while varying the relative timing between the target and flanker faces. As previously reported, we found robust gender congruency effects, namely slower responses to the target faces whose gender was incongruent with that of the flanker faces, when the flankers preceded the target by 0.1 s. When the flankers further advanced the target by 0.3 s, however, the congruency effect vanished in most of the viewing conditions, except for when emotion was expressed only in the flanking faces or when congruent emotion was expressed in the target and flanking faces. These results suggest that emotional saliency can prolong a substantial degree of conflict by diverting bottom-up attention away from the target, and that inhibitory control on task-irrelevant information from flanking stimuli is deterred by the emotional congruency between target and flanking stimuli.

## Introduction

Emotion expressions are crucial for survival, allowing others to immediately infer one’s own internal state via various facial or bodily expressions. Thus, from the perspective of an observer, it is equally important to sense and decipher those signs quickly to infer the agent’s state and react to the environment accordingly. Furthermore, Darwin (1872) was of the opinion that, in the daily experience of perceiving others’ emotion expressions, we automatically attend to those expressions, as pointed out in his remark:

“*Our sympathy being easily aroused when we behold any strong emotion, and our attention thus distracted.*”

This suggests that reacting to someone else’s emotion expression can be either beneficial or costly to sensory encoding of external objects depending on the location of the emotion expressions relative to those of the objects. For instance, emotionally salient or emotion-provoking images are detected more effectively and look more vivid when embedded in other images or visual noise, compared to other non-emotional but equally salient images (Ohman et al., 2001; Vuilleumier et al., 2001; Pinkham et al., 2010; Hodsoll et al., 2011; Todd et al., 2012). While these results indicate that emotion expression has beneficial effects when the object expressing emotion is the target of attention, other studies have found that emotionally arousing stimuli hamper the perception of peripheral stimuli, both in space or time (Most et al., 2005; Blair et al., 2007; Ciesielski et al., 2010; Kennedy and Most, 2012). Together, objects expressing emotion can affect sensory encoding at the expense of objects nearby.

The impact of emotion on cognitive faculties beyond sensory encoding has also been of interest, especially for the faculties involved in goal-directed and executive functions, often referred to as ‘cognitive control’ (Miller, 2000; Miller and Cohen, 2001). Cognitive control comprises many different executive functions including selective attention, inhibitory control, conflict monitoring and motor planning (Mirabella, 2014). One common strategy to study the impact of emotion on cognitive control is to use the tasks involving ‘conflict processing’ such as the Eriksen flanker task (Fenske and Eastwood, 2003; Dennis et al., 2008; Kanske and Kotz, 2010; van Steenbergen et al., 2010; Birk et al., 2011; Schmidt and Schmidt, 2013; Sussman et al., 2013; Zhou and Liu, 2013), the Stroop task (Blair et al., 2007; Hart et al., 2010; Padmala et al., 2011; Zinchenko et al., 2015), the Simon task (Kanske and Kotz, 2011; Melcher et al., 2012), the stop signal task (Verbruggen and De Houwer, 2007; Sagaspe et al., 2011; Pessoa et al., 2012; Kalanthroff et al., 2013), and the go/no-go task (Zhang and Lu, 2012; Yu et al., 2014). When emotion-inducing features were manipulated in those tasks, emotion modulated the processing of conflict resolution. However, it is still unclear whether emotional processing facilitates or deteriorates processing of conflict resolution because presentation of images with negative or positive emotions, such as fearful or happy faces, resulted in either a deterioration (Blair et al., 2007; Verbruggen and De Houwer, 2007; Dennis et al., 2008; Hart et al., 2010; Padmala et al., 2011; Sagaspe et al., 2011; Kalanthroff et al., 2013; Zhou and Liu, 2013) or improvement (Kanske and Kotz, 2010, 2011; van Steenbergen et al., 2010; Birk et al., 2011; Melcher et al., 2012; Zhang and Lu, 2012; Zinchenko et al., 2015).

Given these mixed results, we set out to explore factors that may modulate the influence of emotion on cognitive conflict processing. When choosing the candidate factors to explore, we paid particular attention to the recent advances in the empirical and theoretical understanding of emotional processing. Emotional processing can be divided into “emotional reactivity” and “emotional regulation” [for a review see (Etkin et al., 2015)]. Emotional reactivity refers to a sequence of processes in which emotional objects are perceived, valued, and acted upon (Rangel et al., 2008; Ochsner and Gross, 2014). Emotion regulation refers to a goal-directed process of initiating, stopping, or modulating the trajectory of emotional reactivity when an emotional reaction is the target of valuation (e.g., ‘good-or-bad-for-me’ judgment) or when conflicting emotional reactions are simultaneously present (Gross, 2015). In addition, recent neuroimaging studies suggest common neural loci involved in cognitive control and emotional processing, and, therefore, an active interplay between those two systems (Shackman et al., 2011; Raz et al., 2012, 2014). Based on this intimate relationship between cognitive control and emotional processing, we reasoned that there could be two possible ways in which emotion expressions can influence cognitive control.

First, a salient emotion expressed in a local region is likely to provoke emotional reactivity, which in turn will prioritize our attention to that region. Thus, we reasoned that cognitive control can be facilitated or deteriorated depending on where an emotion-inducing stimulus is located in space relative to a task-relevant target; emotion-inducing features may improve cognitive control if they appear in the same location as the target of a cognitive task and may deteriorate cognitive control if they appear in non-target locations. For this reason, unlike previous studies in which an emotion-inducing stimulus was non-specifically presented in terms of spatial attention (Dennis et al., 2008; Kanske and Kotz, 2010; Birk et al., 2011; Zhou and Liu, 2013; Zhou et al., 2016) we embedded task-irrelevant emotional features either into a spatially attended region within which a task-relevant object appeared, or into spatially unattended regions within which task-irrelevant objects appeared.

Second, we reasoned that conflicting emotion expressions might be detected by the conflict-monitoring system, which in turn promotes regulatory activities for cognitive conflict resolution along the various stages of goal-directed action, such as selective attention or inhibitory control of the information inducing the incongruence. By contrast, we reasoned that the absence of emotional conflicts might encourage the integration of information flows between target and non-target objects. Given the crucial roles of conflict monitoring in cognitive control (Botvinick et al., 2001, 2004; Egner and Hirsch, 2005; Jeffrey Cockburn, 2011), this conjecture suggests that cognitive conflict processing might be facilitated by emotionally conflicting facial expressions but deteriorated by emotionally congruent expressions. To test this possibility, we included a set of viewing conditions where task-irrelevant emotional conflicts were manipulated.

In addition, we hypothesized that the influence of emotion expression on cognitive control might depend on the relative timing differences between cognitive control and emotional processing. For instance, because full-blown neural reactions to emotionally salient stimuli are likely to develop and last over time through a cascade of unfolding processes in the human brain (Gross, 2015), emotional consequences may change over the time course of cognitive control (Schmidt and Schmidt, 2013). While previous studies have typically presented emotion-inducing objects at a fixed time relative to a target (Fenske and Eastwood, 2003; Kanske and Kotz, 2010; Lichtenstein-Vidne et al., 2012), we varied the relative timing of task-irrelevant emotional features to task-relevant features.

To manipulate these three factors, we adopted the Eriksen flanker paradigm (Eriksen and Eriksen, 1974) and used facial images as stimuli. Participants viewed displays of a central facial image flanked by peripheral facial images and judged the gender of the central face as accurately and fast as possible. Since the information stemming from the flanking faces with the gender opposite to that of the target face will lead to conflicting responses, an observer needs to resolve this conflict by exploiting ‘spatial selective attention’—focusing attention to a central object—and ‘inhibitory control’—actively ignoring competing objects in the periphery. The Eriksen flanker paradigm allows us to estimate the behavioral cost of resolving this conflict quantitatively by the ‘gender congruency effect,’ which is the measure of the delay of gender judgments when the gender was incongruent between the target and the flankers compared to when it was congruent. The amount of gender congruency effect will increase or decrease depending either on the degree of conflict or on the time or effort devoted by the executive system that resolves such conflict. To study how emotional processing affects cognitive conflict processing, we carried out three experiments by measuring the gender congruency effect while manipulating the emotion expression of the facial images and the stimulus onset asynchrony (SOA) between the target and flankers. In this way, the task-relevant features, which contribute to gender discrimination, and the task-irrelevant features, which are associated with emotion expression, could be manipulated within a single stimulus. In the following paragraphs, we provide the motivation, rationale and conditions for each of the three experiments in detail.

In Experiment 1, participants made gender judgments of a central face image while task-irrelevant emotion expression was embedded either in the target face only (the ‘emotion in target’ condition) or in the flanker faces only (the ‘emotion in flankers’ condition). This spatial manipulation of task-irrelevant emotional features allows us to resolve the influence of emotion on cognitive control of selective attention. Specifically, we hypothesized that goal-directed selective attention would be expected to be facilitated in the ‘emotion in target’ condition because an emotionally salient face draws bottom-up attention to the central target due to emotional attention capture but is deteriorated in the ‘emotion in flankers’ condition because an emotionally salient face draws bottom-up attention to the peripheral flankers. To test this hypothesis, we compared the gender congruency effects between the ‘emotion in target’ and the ‘emotion in flankers’ conditions over the three different ‘target-flanker SOA’ conditions.

In Experiment 2, we manipulated the color tone, instead of emotion expression, of the facial images. The robust gender congruency effect was found when emotion was expressed only in the flanking faces that advanced the target by 0.3 s in Experiment 1. To know whether this prolonged interruption of cognitive control can be ascribed specifically to emotion expression only or generally to other non-emotional yet perceptually salient features as well, we carried out Experiment 2, which was identical to Experiment 1 except that primary colors were used as a task-irrelevant feature instead of emotion expressions. We used primary colors because a lone colorful object in a homogenous field looks highly salient and immediately attracts attention, no matter how many other objects are presented in the field (Treisman and Gelade, 1980; Wolfe, 1998). If salience alone explained the findings of Experiment 1, the same location-dependent pattern of congruency effects could be expected in Experiment 2.

In Experiment 3, we manipulated “emotional conflict” by presenting congruent or incongruent emotion expressions between the target and flanking faces. Recent studies suggest that an agent flexibly regulates cognitive control by executing or canceling goal-directed actions depending on the future benefits and costs of those actions (Mirabella, 2014; Botvinick and Braver, 2015). By capitalizing on this link between the factors that regulate cognitive control such as sensory conflicts and execution/cancelation of cognitive control, we explored the possibility that the presence or absence of “emotional conflict” between the target and the flankers, despite being task-irrelevant, affects the execution of cognitive control. For this, emotion expressions were present both in the target face and in the flanking faces, and we manipulated the congruency in emotional valence between them. We hypothesized that if conflict in emotional expression, even when task-irrelevant, is detected by the conflict-monitoring system and thus facilitates inhibitory control of task-irrelevant feature processing, then the behavioral cost of conflict resolution—gender congruency effect—would be lower in the ‘incongruent in emotion’ condition than in the ‘congruent in emotion’ condition.

## Materials and Methods

### Participants

A total of 62 paid (approximately 8.4 USD/hour) undergraduate or graduate students participated in one of three experiments. A total of 20 (nine females, aged 20–26 years), 20 (nine females, 19–32 years), and 22 (11 females, 19–29 years) students participated in Experiments 1, 2, and 3, respectively. Two participants in Experiment 3 were excluded from further analyses because they fell asleep or hummed songs during the experiment. All participants were naïve as to the purpose of the experiments. They had normal or corrected normal vision and participated in only one of the three experiments. The study was approved by the Seoul National University Institutional Review Board, and informed written consent was obtained from each participant before the experiments.

### Stimuli and Apparatus

**FIGURE 1:**
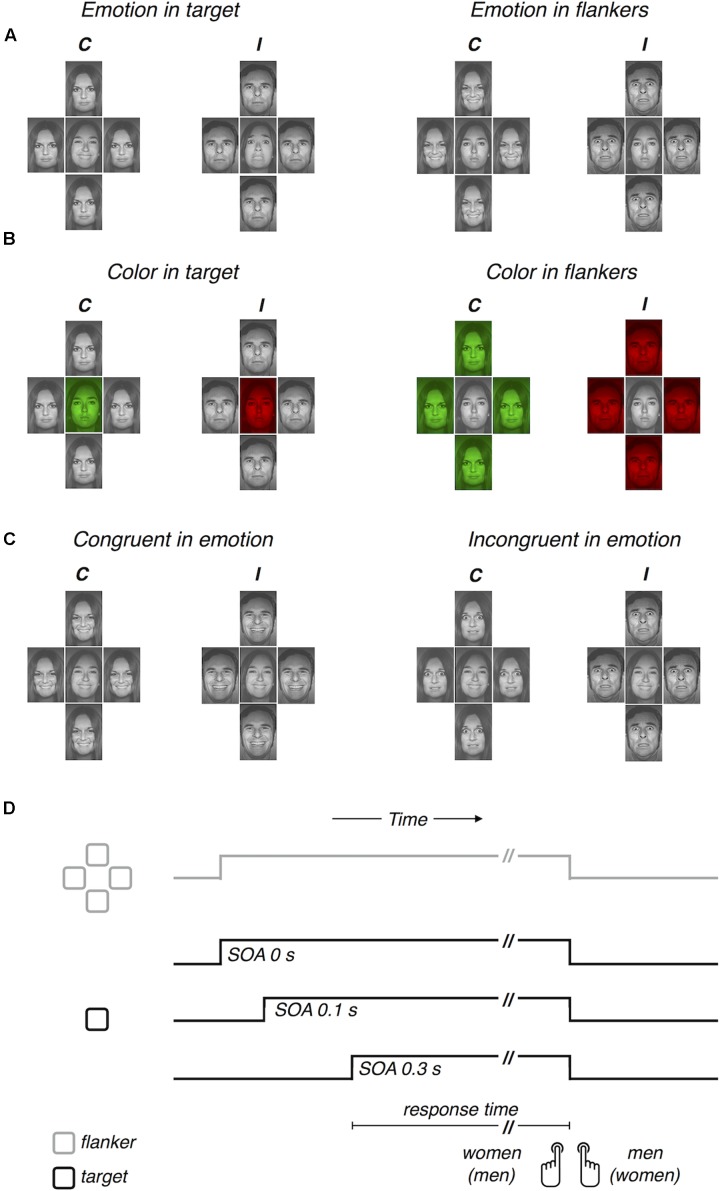
Task, stimuli, viewing conditions, and procedure. The task was to discriminate the gender of the face at the center, ∼ which was identical to (gender congruent condition, labeled as ‘*C*’), or different (gender incongruent condition, labeled as ‘*I*’) from that in the periphery. **(A)** Viewing conditions manipulated in Experiment 1, where task-irrelevant emotion was expressed either in the target or in the flankers. **(B)** Viewing conditions manipulated in Experiment 2, where task-irrelevant color tone was manipulated either in the target or in the flankers. **(C)** Viewing conditions manipulated in Experiment 3, where task-irrelevant emotion was congruent or incongruent between the target or in the flankers. **(D)** Schematic procedure illustrating the time course of the target (single black square) and the flankers (four gray squares) for three SOA conditions (0, 0.1, and 0.3 s).

Visual stimuli were 24 face images from eight people (four women and four men), expressing “happy,” “fearful,” and “neutral” facial emotions. These images were selected from a facial expression database, the Pictures of Facial Affect (Ekman and Friesen, 1976), and permission to use them was obtained from the Paul Ekman Group, LLC^1^. Participants viewed a display, where a central target image was flanked vertically and horizontally by four distractor images, and classified the target face as either “male” or “female” (**Figures 1A–C**). The target and the flankers always differed in facial identity, while the four flankers were always identical to one another in terms of both identity and facial expression. All images were adjusted to match in size (a rectangle of 1.84° × 1.58° of visual angle), in mean luminance (44.18 cd/m^2^), and in image contrast (15.7% root mean square contrast). The target and flanker images were separated from one another by a gap of 0.05°. The images were presented in monotone color scale (gray scale for Experiments 1 and 3; green, red, and gray scale for Experiment 2) against a dark (0.32 cd/m^2^) background. Visual stimuli were generated by running the Psychophysics Toolbox extensions (Brainard, 1997; Pelli, 1997; Kleiner et al., 2007) in conjunction with MATLAB 2014b (The MathWorks Inc.) on an iMac computer and displayed on a separate 19-inch LCD monitor (DELL 1905 FP) with a spatiotemporal resolution of 1280 pixels × 1024 pixels and 60 Hz in a dark room. The monitor was 65 cm away from participants’ eyes.

### Procedure

In each trial, participants gazed at the center of the display, where a small (0.75°) fixation cross appeared at the trial onset and stayed for 0.7–1.0 s until presentation of the target image. To see whether and how the emotional influence on cognitive control depends on the relative timing differences between cognitive control and emotional processing, we varied the onset of flanker images to be either earlier or equal to that of the target image, resulting in three conditions of SOA (0-s, 0.1-s, and 0.3-s SOAs; **Figure 1D**). Participants were instructed to judge the gender of the target image as accurately and quickly as possible and to respond by pressing one of two keys on a keyboard. The target and flanker images both remained displayed until participants made a response or 1.5 s from the target onset. Thus, the flanker images never disappeared before the onset of the target image, accompanying the target image always until the moment of button press, regardless of the SOA conditions. After the offset of the stimuli, the entire screen went blank for variable durations (0.3–0.7 s) before the next trial began.

In all experiments, the task-relevant information was conjunctively defined in two domains. In the space domain, the task-relevant information was provided at the central position. In the feature domain, the task-relevant information was the ensemble of facial features contributing to gender discrimination. Thus, for fast yet accurate gender discrimination of the central target, which was flanked by the peripheral distractors, participants had to integrate the task-relevant information by deploying their selective attention to the gender-discriminating features of a facial image presented at the target location. As in previous studies, the efficiency of selective information processing was estimated by measuring how much gender discrimination of the target was delayed when the distractors carried conflicting gender information compared to when they did not, ‘congruency effect.’ In the current study, we further distracted selective information processing by additionally manipulating the task-irrelevant features of facial images in various ways. In Experiments 1 and 2, we examined how the spatial location of task-irrelevant features affects the processing of the task-relevant features by manipulating the task-irrelevant feature either only in the target image or only in the flanker images. The task-irrelevant feature was “emotion expression” in Experiment 1 (**Figure 1A**) and “color tone” in Experiment 2 (**Figure 1B**). In Experiment 3, we manipulated the task-irrelevant emotion expression both in the target and flanker locations simultaneously (**Figure 1C**).

All experiments included the same three SOAs (**Figure 1D**). We opted to use this particular set of SOAs because the congruency effect is expected to vary dynamically over this range, reaching its peak at around a 0.1-s SOA (Taylor, 1977; Flowers and Wilcox, 1982; Flowers, 1990; Schmidt and Schmidt, 2013). Thus, this range of SOAs was expected to sufficiently detect any potential effects of task-irrelevant emotion expression. In addition, using three SOAs allowed us to explore the time-dependent changes of any interactions between task-relevant cognitive control and task-irrelevant emotion reactivity (and its regulation). Below, we described the detailed procedures specific to each experiment.

#### Procedure for Experiment 1

The viewing conditions in Experiment 1 were manipulated by three independent variables: (1) ‘congruency in facial gender,’ (2) ‘target-flanker SOA,’ and (3) ‘location of emotion expression.’ The ‘congruency in facial gender’ had two conditions: congruent or incongruent target and flankers. The ‘target-flanker SOA’ had three conditions: 0, 0.1, and 0.3 s (**Figure 1D**). The ‘location of emotion expression’ had two conditions, each comprising two separate blocks of trials, the order of which was counterbalanced across participants. In the ‘emotion in target’ blocks (left two panels of **Figure 1A**), the facial expression varied, being “happy,” “fearful,” or “neutral,” across trials only in the target region but remained “neutral” across trials in the flankers. The target and the flankers had different identities, but the four flankers had the same identity (e.g., the “happy” face of a person was flanked by the four same “neutral” faces of another person). In the ‘emotion in flanker’ blocks (right two panels of **Figure 1A**), the facial expression varied only in the flankers and remained “neutral” in the target (e.g., the “neutral” face of a person was flanked by the four same “fearful” faces of another person).

Each block consisted of 90 trials, yielding a total of 1,800 trials (two block types × 10 blocks × 90 trials) and 150 trials for each of the 12 viewing conditions (two ‘congruency in facial gender’ conditions × three ‘target-flanker SOA’ conditions × two ‘location of emotion expression’ conditions). All possible combinations of variables not of main interest (e.g., facial identity) were counterbalanced and randomized. Before the first block for each type, participants completed 30 practice trials and received trial-by-trial feedback. Participants only received feedback about the overall accuracy (the fraction of correct trials) after each block. Key assignment was counterbalanced across participants.

#### Procedure for Experiment 2

Procedures were identical to those in Experiment 1 except for that the color tone of facial images was manipulated as the task-irrelevant feature (**Figure 1B**). Specifically, the facial expressions of “happy,” “fearful,” and “neutral” were replaced by the color tones of “green,” “red,” and “gray” (compare the facial images at corresponding locations in **Figures 1A,B**).

#### Procedure for Experiment 3

The viewing conditions in Experiment 3 were manipulated in a manner similar to that for Experiment 1. The two independent variables, (1) ‘congruency in facial gender’ and (2) ‘target-flanker SOA,’ were also used and manipulated in the same way as in Experiments 1 and 2. Instead of manipulating the ‘location of emotion expression,’ however, we introduced a new variable, (3) ‘congruency in emotion expression.’ This new variable had two conditions. In the “congruent in emotion” condition (left two panels of **Figure 1C**), the target and the flankers were matched in emotion expression, being all either “happy” or “fearful.” In the ‘incongruent in emotion’ condition (right two panels of **Figure 1C**), the emotion expression differed between the target and the flankers. Unlike Experiment 1, “neutral” faces were not used at all, and the trials of ‘congruent in emotion’ and ‘incongruent in emotion’ conditions were not separated between blocks but randomly intermixed within single blocks of trials. Experiment 3 consisted of 12 consecutive blocks of 100 trials, resulting in a total of 1200 trials and 100 trials for each the 12 viewing conditions (two ‘congruency in facial gender’ conditions × three ‘target-flanker SOA’ conditions × two ‘congruency in emotion expression’ conditions). Participants completed 30 practice trials before the main trials. The procedures regarding feedback and response key assignment were identical to those for Experiments 1 and 2.

### Data Preprocessing and Statistical Analysis

**FIGURE 2:**
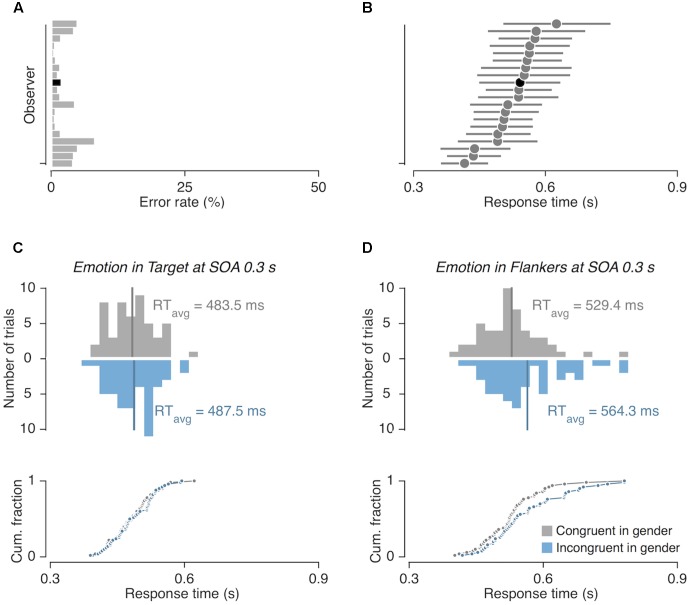
Computation of congruency effects from response time (RT) measurements. **(A)** Error rates and **(B)** mean RTs of correct responses shown for 20 individual participants in Experiment 1. The horizontal lines in **(B)** demarcate the 84% range of RT distribution. The black bar and circle in **(A,B)** represent the error rate and RT measurements from a representative participant, whose example data are shown in **(C,D)**. **(C,D)** Histograms (top panels) and cumulative distributions (bottom panels) of RTs shown for two example viewing conditions. Congruency effects were computed by subtracting the mean RT of gender-congruent trials (gray vertical lines) from that of gender-incongruent trials (blue vertical lines).

Across all experiments, participants rarely made incorrect gender judgments [mean error rates for Experiment 1 (**Figure 2A**) = 2.63%; Experiment 2 = 2.07%; Experiment 3 = 2.01%]. Thus, we analyzed response time (RT) data acquired from the trials in which participants made correct judgments (**Figure 2B**). Data from trials in which RTs were extremely fast or slow were discarded from further analysis; valid RT measures were defined as those falling within the ±3 standard deviations from the RT averaged across all correct trials for each participant and all experiments. This resulted in an exclusion of 1.75%, 1.79%, and 1.73% of correct trials for Experiments 1, 2, and 3, respectively.

To evaluate the contributions of the task-irrelevant features and the SOAs to the congruency effects in RT measures, we conducted statistical analyses as follows. First, we ran repeated-measures analyses of variance (ANOVAs) to test whether significant RT differences existed between the factors of interest. Results of ANOVAs were reported with *F*-values, Sphericity Assumed *p*-values, and the effect size ($\eta_{p}^{2}$; Cohen, 1973).

If this ANOVA returned a significant result, the Bonferroni test was used to perform *post hoc* evaluations of pairwise differences between the factors to compare the congruency effects between the conditions of task-irrelevant feature manipulation at each of the SOA conditions. Bonferroni-adjusted *p*-values were used to judge the significance of *post hoc* comparisons, unless stated otherwise.

## Results

### Experiment 1: Emotional Saliency in Flanking Faces Prolongs Congruency Effects

Across the participants, the mean overall RT was slower when the gender was incongruent (532 ms ± 11.2 ms) than when it was congruent (519 ms ± 12.1 ms) [*F*_congruency_ (1,19) = 26.492, *p* < 0.001, $\eta_{p}^{2}$ = 0.853]. Having confirmed the overall gender congruency effect (i.e., the delayed RT to the gender-incongruent faces; **Figures 2C,D**), we examined whether the degree of effect was modulated depending on the other factors.

First, the size of congruency effect varied substantially depending on the SOAs’ length [*F*_Congruency × SOA_ (2,38) = 17.840, *p* < 0.001, $\eta_{p}^{2}$ = 0.484]. The congruency effect was almost negligible when the target and flankers were presented simultaneously (0-s SOA; 2.61 ms ± 1.44 ms), reached its maximum when the flankers advanced the target by 0.1 s (0.1-s SOA; 20.31 ms ± 3.48 ms), and then diminished when the flankers advanced the target by 0.3 s (0.3-s SOA; 12.77 ms ± 3.27 ms).

**FIGURE 3:**
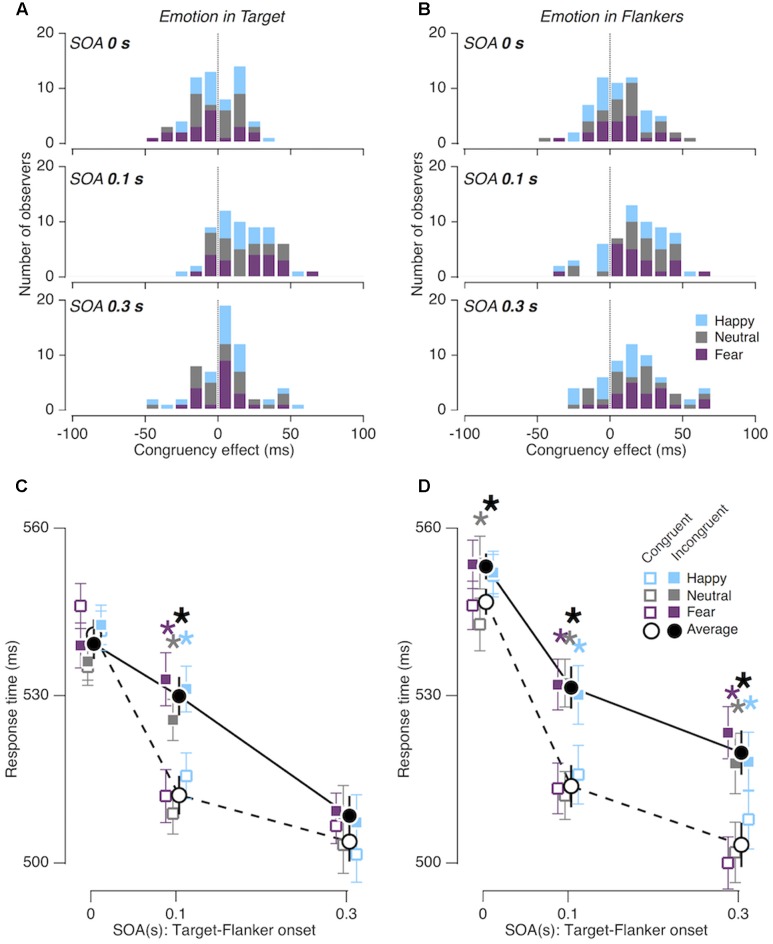
Congruency effects in Experiment 1. **(A,B)** Distributions of congruency effects from the ‘emotion in target’ condition **(A)** and the ‘emotion in flankers’ condition **(B)**, shown for the three different SOA conditions. **(C,D)** Comparisons of mean RTs between gender-congruent trials (empty symbols and dashed line) and gender-incongruent trials (solid symbols and solid line) for the ‘emotion in target’ condition **(C)** and for the ‘emotion in flankers’ condition **(D)**. Error bars represent 95% confidence interval of standard error. Asterisks, statistically significant (*p* < 0.05) data points.

In addition, we also assessed whether this modulation was affected by the location of task-irrelevant emotion expression. The pattern of SOA-dependent congruency effects indeed differed depending on whether the task-irrelevant emotion expression was embedded in the central face (the ‘emotion in target’ condition; **Figures 3A,C**) or in the peripheral faces (the ‘emotion in flankers’ condition; **Figures 3B,D**) [*F*_Congruency × SOA × Location_ (2,38) = 3.691; *p* = 0.034; $\eta_{p}^{2}$ = 0.163]. *Post hoc* tests showed that the congruency effect was significantly greater in the ‘emotion in flankers’ condition than in the ‘emotion in target’ condition at the SOAs of 0 and 0.3 s (*p* = 0.034; $\eta_{p}^{2}$ = 0.216 and *p* = 0.012; $\eta_{p}^{2}$ = 0.291, respectively; **Figures 3C,D**). This indicates that gender judgments in the gender-incongruent trials were slower when the task-irrelevant emotion was expressed in the flanker faces than when it was expressed in the target face. Nevertheless, this three-way interaction effect between ‘congruency in facial gender,’ ‘target-flanker SOA,’ and ‘location of emotion expression’ factors was not modulated by the valence of emotion expression [“happy,” “fearful,” “neutral”; *F*_Congruency × SOA × Location × V alence_ (4,76) = 1.064; *p* = 0.380; $\eta_{p}^{2}$ = 0.053].

There was a block-dependent congruency effect in the “neutral” trials, in which the facial expression was neutral both in the target and the flankers. Although these trials were physically identical, the congruency effect at 0.3-s SOA was significant in the ‘emotion in flankers’ blocks, but non-significant in the ‘emotion in target’ blocks.

### Experiment 2: Color Saliency Does Not Prolong Congruency Effects

**FIGURE 4:**
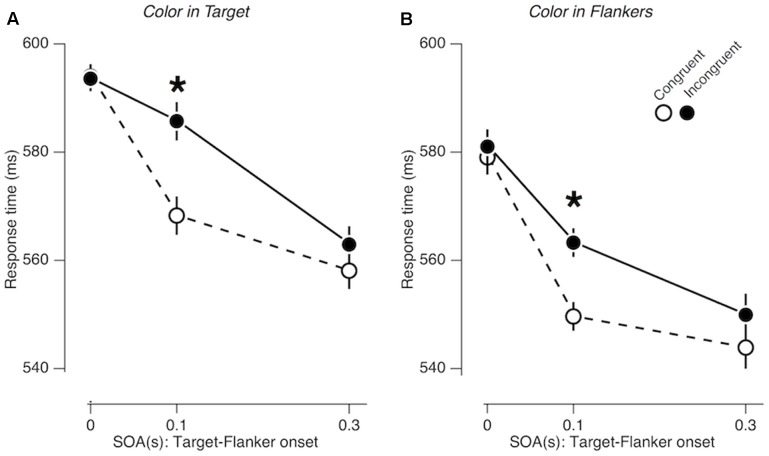
Congruency effects in Experiment 2. **(A,B)** Comparisons of mean RTs between gender-congruent trials and gender-incongruent trials for the ‘color in target’ condition **(A)** and for the ‘color in flankers’ condition **(B)**. The format is identical to that in **Figures 3C,D**.

As in Experiment 1, the gender congruency effect was significant overall [*F*_Congruency_ (1,19) = 14.840; *p* = 0.001; $\eta_{p}^{2}$ = 0.439], and its size varied substantially as a function of SOA [*F*_Congruency × SOA_ (2,38) = 10.654; *p* < 0.001; $\eta_{p}^{2}$ = 0.359]. Unlike Experiment 1, however, the pattern of SOA-dependent congruency effects remained the same, regardless of whether the task-irrelevant color features accompanied the central face (‘color in target’ condition; **Figure 4A**) or the peripheral faces (‘color in flankers’ condition; **Figure 4B**) [*F*_Congruency × SOA × Location_ (2,38) = 1.353; *p* = 0.271; $\eta_{p}^{2}$ = 0.066]. The congruency effect was significant only at the SOA of 0.1 s both in the ‘color in target’ condition and in the ‘color in flankers’ condition (*p* < 0.001; $\eta_{p}^{2}$ = 0.590 and *p* < 0.00; $\eta_{p}^{2}$ = 0.639,respectively).

The results of Experiment 2 indicated that the congruency effects at the SOA of 0.3 s found in Experiment 1 could not be simply attributed to saliency of task-irrelevant features. Instead, our results suggest that the influence of emotion expression on cognitive control goes beyond what can be inflicted by low-level, salient visual features, such as transient, bottom-up distraction of attention.

### Experiment 3: Emotion Congruency between Target and Flankers Prolongs Gender Congruency Effects

**FIGURE 5:**
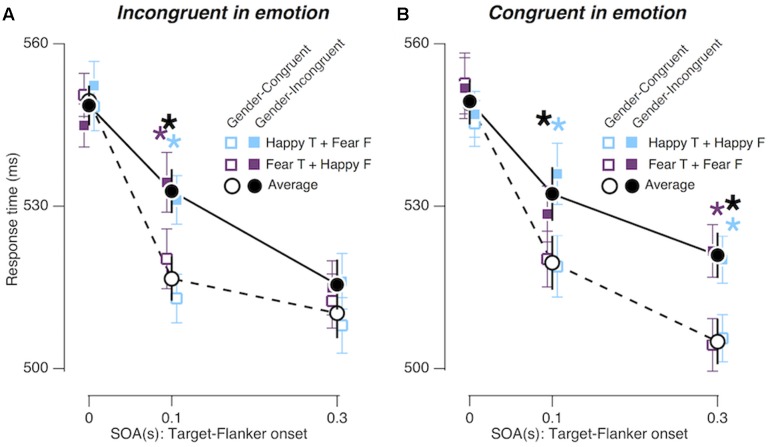
Congruency effects in Experiment 3. **(A,B)** Comparisons of mean RTs between gender-congruent trials and gender-incongruent trials for the ‘incongruent in emotion’ condition **(A)** and for the ‘congruent in emotion’ condition **(B)**. The format is identical to that in **Figures 3C,D**.

We confirmed the overall congruency effect [*F*_Congruency_ (1,19) = 9.673; *p* = 0.006; $\eta_{p}^{2}$ = 0.337] and its SOA-dependent changes [*F*_Congruency × SOA_ (2,38) = 6.687; *p* = 0.003; $\eta_{p}^{2}$ = 0.260], which were found in Experiments 1 and 2. More importantly, the gender congruency effect differed between the ‘congruent in emotion’ and the ‘incongruent in emotion’ conditions over different ‘target-flanker SOA’ conditions (**Figure 5**), as indicated by the marginal interaction effect between gender congruency, SOA, and emotion congruency [*F*_Congruency × SOA × EC_ (2,38) = 2.996; *p* = 0.062; $\eta_{p}^{2}$ = 0.136]. Specifically, when the flankers advanced the target by 0.3 s, the congruency effect was significant in the congruent emotion condition, but not significant in the incongruent emotion condition (*p* = 0.001; $\eta_{p}^{2}$ = 0.446 and *p* = 0.255; $\eta_{p}^{2}$ = 0.068, respectively).

### Summary of the Results from Experiments 1, 2, and 3

**FIGURE 6:**
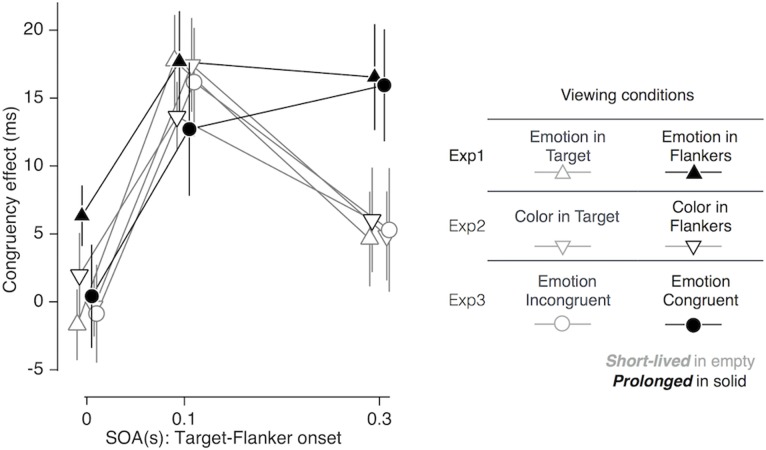
Comparisons of congruency effect across all viewing conditions as a function of SOA. Error bars represent 95% confidence interval of standard error. See the right panel for the legends for the symbols and lines.

When the flankers appeared ahead of the target by 0.1 s, the gender congruency effect was observed consistently in all viewing conditions examined in our study. When the flankers further advanced the target by 0.3 s, however, the congruency effect disappeared in most of the conditions but remained significant only in the following two conditions (solid symbols in **Figure 6**): (i) when emotion expression was embedded only in the flankers [*F*(1,19) = 18.240; *p* < 0.001; $\eta_{p}^{2}$ = 0.490; ‘emotion in flankers’ condition of Experiment 1; solid triangles in **Figure 6**], and (ii) when emotion expression of the flankers matched that of the target [*F*(1,19) = 15.302; *p* = 0.001; $\eta_{p}^{2}$ = 0.446; ‘congruent in emotion’ condition of Experiment 3; solid circles in **Figure 6**]. The prolonged congruency effects in these two conditions were invariant to the gender and valence of facial images. As a control, we confirmed that the congruency effect found at the SOA of 0.3 s in the ‘emotion in flankers’ condition did not hold when non-emotional but highly salient low-level features, i.e., monotonic tones of primary colors, were used as task-irrelevant features (‘color in flankers’ condition of Experiment 2; black empty downward triangles in **Figure 6**). This indicates that the sustained congruency effect in the ‘emotion in flankers’ condition cannot be simply explained by the low-level saliency effect known to capture bottom-up attention (Treisman and Gelade, 1980).

## Discussion

Emotional reactivity refers to a sequence of processes in which emotional objects are detected, evaluated, and translated into actions. The goal-directed adjustment of emotional reactivity, dubbed “emotional regulation,” occurs either when emotionally salient objects induce emotional reactions that would interfere with task-oriented activity or when there is conflict in valence between emotional reactions (Etkin et al., 2011, 2015). Recent neuroimaging studies support this view by showing that the cognitive control and emotion processing have overlapping cortical systems (Shackman et al., 2011; Raz et al., 2012, 2014). Guided by this perspective, we embedded the emotion expression into facial images to provoke task-irrelevant emotional reactivity and created the two critical situations known to instigate or promote emotional processing. In Experiment 1, we manipulated the location of emotional saliency by provoking emotional reactivity either in the target face or in the flanking faces. In Experiment 3, we manipulated the presence or absence of emotional conflict as a means of regulating emotional reactivity. We then examined how these manipulations affected cognitive control of conflict processing by comparing congruency effects using the Eriksen flanker paradigm (Eriksen and Eriksen, 1974).

Below, we present a comprehensive and coherent discussion of our findings in threefolds. First, the location-dependent emotional influences on the gender congruency effect (Experiment 1; upright triangles in **Figure 6**) imply that emotionally salient facial expressions in a task-irrelevant region can trigger the emotional reactivity of prioritizing bottom-up attention to that region and thus counteract the task-relevant spatial selective attention. Second, the effects of emotional congruency on the gender congruency effect (Experiment 3; circles in **Figure 6**) imply that the presence of congruent emotional expressions between target and flankers can deter the execution of inhibitory control. Finally, the prolongation of emotional interruption of cognitive control, i.e., the substantial congruency effects found up to 0.3 s after the onset of flankers, may reflect the fact that it takes time for both emotional processing and cognitive control to become fully effective and thus interact with each other; for instance, via a cascade of unfolding processes (Gross, 2015) and gradual build-up of selective suppression (Ridderinkhof, 2002; Ridderinkhof et al., 2004), respectively.

### Emotion Expression in Flankers Counteracts Spatial Selective Attention to Gender Information in a Target

Experiment 1 found that the gender congruency effect was more prolonged when task-irrelevant emotional features were embedded in the flanking faces. Why was the behavioral cost of conflict processing higher in these conditions? One may refer to the well-known findings that facial images with emotional expressions are salient enough to capture bottom-up attention, thus evoking strong neural responses (Vuilleumier et al., 2001). However, this does not fully explain why the congruency effect at the SOA of 0.3 s was not observed when the primary colors, i.e., salient, but not emotional features, were applied to the face images in the flanking regions in Experiment 2. If the capture of bottom-up attention by perceptually salient distractors were alone responsible, then no such difference in the congruency effect would have been observed. Thus, “perceptual saliency” is not sufficient to explain these findings, and “emotional features” seem necessary for the prolonged congruency effect by the flankers. This implies that emotionally salient stimuli, compared to non-emotionally salient stimuli, are more difficult to be controlled by top-down selective attention. This implication seems consistent with previous studies on brain-damaged patients, which suggested that facial or bodily emotion expressions are processed pre-attentively or automatically (Vuilleumier and Schwartz, 2001; Vuilleumier et al., 2002; Anders et al., 2009; Tamietto et al., 2015). For instance, the amygdala and orbitofrontal cortex—areas known for representing emotional reactivity—were readily activated by emotional faces even in patients with hemispatial neglect and visual extinction due to damaged right inferior parietal cortex (Vuilleumier and Schwartz, 2001). In addition, GY, a patient with damaged primary visual cortex, could discriminate facial expressions presented in his blinded visual field above a chance level (De Gelder et al., 1999), a phenomenon called “affective blindsight” [see Celeghin et al. (2015) for a review].

As mentioned earlier, the non-specific influence of emotion on cognitive control might either be always deteriorative (Verbruggen and De Houwer, 2007; Dennis et al., 2008; Hart et al., 2010; Padmala et al., 2011; Kalanthroff et al., 2013) or always facilitatory (Kanske and Kotz, 2010, 2011; Birk et al., 2011; Melcher et al., 2012; Zinchenko et al., 2015). However, this conjecture is at odds with our finding of location-specific modulation of the prolonged congruency effect at the SOA of 0.3 s (as visualized by the contrast between the empty and solid triangles in **Figure 6**). Instead, we prefer to interpret this prolonged gender congruency effect found in the ‘emotion in flankers’ condition as a counteraction of bottom-up emotion-induced imbalance in spatial attention against goal-directed execution of top-down spatial selective attention. Facial expressions with strong emotional valence, such as those used in the current study, are likely to promote substantial emotional reactions and subsequent unfolding emotional processes not only for those embedded in the cognitively attended, target face, but also for those in cognitively unattended, peripheral faces.

As mentioned in section “Experiment 1: Emotional Saliency in Flanking Faces Prolongs Congruency Effects”, in trials in which the target and the flankers were all neutral faces, the congruency effect at the SOA of 0.3 s was observed when those trials were intermixed with the trials with emotional flankers, but not when intermixed with those with an emotional target. What could have resulted in these “within-block” congruency effects for the neutral faces? We considered two possible candidates: “emotion unfolding effects” and “emotion aftereffects.” Emotion unfolding effects refer to the possibility that the emotional reactions are not triggered on and off swiftly in a moment-to-moment fashion, but instead unfold gradually, thus sustaining over an extended period of time. In other words, emotional reactions induced by emotional facial expressions might have lingered over several trials, and may have therefore been capable of counteracting the cognitive control of selective attention. This interpretation is only speculative, but certainly not implausible given the iterative and accumulative nature of emotion regulation (Gross, 2015). Alternatively, the emotion aftereffects (Webster et al., 2004), i.e., the tendency to perceive an emotion expression (e.g., “happy”) that is the opposite of that of an adapted emotion (e.g., “fearful”), suggests that visualization of neutral faces after the visualization of an emotional face in a previous trial can lead to them being perceived as emotional faces. In other words, neutral flankers could have acted as pseudo fearful flankers after happy flankers appeared in previous trials, or as pseudo happy flankers after fearful flankers. Although the emotion unfolding effects and the emotion aftereffects differ in time scale, the former lingering over several trials and the latter occurring on a trial-to-trial basis, both explanations are equally plausible, and indistinguishable in the current experimental design. To resolve this issue, further experiments would be required, in which the two factors, carry-over and adaptation, are orthogonally manipulated.

### Emotion Expression Congruent between Target and Flankers Hinders Inhibitory Control

Cognitive control can be regulated in a flexible manner at any moments of time until goal-directed actions are completed by evaluating the future benefits and costs of those actions (Mirabella, 2014; Botvinick and Braver, 2015) or by monitoring conflicts of diverse origins, response errors, or novel events (Dixon and Christoff, 2012; Cavanagh and Frank, 2014). When interpreted in the emotion regulation framework, previous neuroimaging results from tasks involving emotion conflict appear to offer an interesting account for our findings of the prolonged gender congruency effect for the target and flankers whose task-irrelevant facial features were matched (congruent) in emotional valence. Using a variant of the Stoop paradigm, Egner et al. (2008) showed that the lateral prefrontal cortex controls non-emotional distractors by enhancing the task-relevant responses to targets, whereas the rostral anterior cingulate cortex controls emotional distractors by decreasing task-irrelevant responses to emotional distractors. Using a variant of the Flanker paradigm, Ochsner et al. (2009) also reported that the emotional (categorization of words into positive vs. negative valences) and non-emotional (categorization of words into metal vs. fruit categories) flanker tasks recruit different cortical areas; the rostral cingulate cortex/ventromedial prefrontal cortex and ventrolateral prefrontal cortex (vlPFC), respectively. Furthermore, Dolcos and McCarthy (2006) demonstrated the interference of facial memory task performance by task-irrelevant emotional scenes, and this interference was accompanied by the activation of a cortical area involved in inhibiting emotion (vlPFC) and the deactivation of a cortical area involved in memory performance (dorsolateral prefrontal cortex). Put together, these neuroimaging studies suggest that the human brain is equipped with two separate mechanisms for controlling emotional and non-emotional stimuli, and that the two mechanisms can interfere with each other when arranged such that one is recruited in task-relevant information processing while the other in task-irrelevant information processing. In this sense, one possible interpretation of the prolonged and augmented gender congruency effects for the emotionally non-conflicting target and flanker faces in Experiment 3 might be that the cortical mechanism for detecting the need for inhibitory control, the presence of sensory conflicts in our case, is hindered by the cortical mechanism for emotional regulation that detected the opposite situation, the absence of conflicts. This is likely to lead the integration of congruent emotional reactions between the target and the flankers. Our interpretation seems consistent with a recent event-related-potential study, which suggests that processing of emotion conflict is modulated by top-down attention in a manner similar to cognitive control (Zhou et al., 2016).

### Prolonged Congruency Effects at 0.3-s SOA

The temporal dynamics of the congruency effects was quite robust, exhibiting an inverted “v”-shape in most of the viewing conditions: very weak, maximal, and then diminishing at the 0-s, 0.1-s, and 0.3-s SOAs, respectively. This confirms and extends the findings by previous studies on the flanker paradigm (Flowers and Wilcox, 1982; Flowers, 1990; Schmidt and Schmidt, 2013). For example, our results replicate those of Flowers and Wilcox (1982), who reported that the congruency effect increased when letter flankers preceded a target letter, and then decreased after reaching the maximum point at around 0.1 s. Schmidt and Schmidt (2013) also demonstrated the inverted v-shape pattern in a flanker experiment using schematic faces, showing that the congruency effect increased when the flanker faces preceded the target face, but decreased when the target preceded the flankers or when the target and flankers appeared simultaneously. The “priming effect” has been considered to account for the inverted v-shape of the congruency effect (Flowers and Wilcox, 1982; Denton and Shiffrin, 2012) because priming is short-lived and thus creates a narrow time window during which the flankers can interfere with the processing of the target. Again, from the perspective of emotion regulation, the prolonged congruency effect in our study suggests that the emotional reaction, presumably promoted in the subcortical and cortical network of emotional reactivity, can be a cascade of unfolding processes unless regulated by the cortical network of emotion regulation (Etkin et al., 2015).

### Limitations and Future Studies

The results reported here should be interpreted with some cautionary notes. First, our participants, who were all Asian, performed the task on the images of Caucasian faces. As shown by previous studies, Asians may perceive Caucasian facial expressions, particularly negative emotions, differently from Caucasians (Elfenbein and Ambady, 2003; Zebrowitz et al., 2010). This could explain why no valence effects were found, which have otherwise been consistently reported in previous studies (Fenske and Eastwood, 2003; Holthausen et al., 2016). Thus, the effect of emotional valence on the congruency effects must be addressed properly in future studies by using the images of faces that have the same ethnic identity as that of the participants. Second, the temporal dynamics of congruency effects, i.e., the inverted v-shape, was quite robust throughout all the experiments, and the time points were carefully chosen based on previous studies (Flowers and Wilcox, 1982; Schmidt and Schmidt, 2013). However, we need to confirm our findings, either over finer time scales or for wider SOA ranges, in future studies, because the time course of congruency effects augmented by task-irrelevant emotional features might turn out to be more complicated or even longer than the longest SOA (0.3 s) currently explored.

We are quick and ready to infer emotional states from the subtle facial features of our neighbors, presumably because this ability helps us to react efficiently to important physical or social events, thus providing us with evolutionary advantages. This implies that even when we are engaged in a demanding cognitive task, emotional expressions, irrespective of their relevance to the task, are likely to trigger emotional reactions. The current study, guided by the recent advance in understanding how the brain processes and regulates such emotional reactions, demonstrated that task-irrelevant emotional expressions can interfere with the cognition of human faces over an extended period of time. We suggest that the spatial imbalance in emotional saliency counteracts cognitive control of selective attention in one route whereas the congruency in emotional valence hinders the execution of inhibitory control in the other route. These two suggested routes, and the temporal dynamics of emotion-cognition interplay occurring there, certainly merit further investigation, particularly with respect to the identification of their neural substrates via neuroimaging or electrophysiological experiments.

## Author Contributions

JK, M-SK, YC, and S-HL contributed to the conception and design of the work. JK acquired the data. JK and S-HL carried out the data analysis. JK and S-HL wrote an initial draft of the manuscript. JK, S-HL, M-SK, and YC contributed to the revision of the manuscript.

## Conflict of Interest Statement

The authors declare that the research was conducted in the absence of any commercial or financial relationships that could be construed as a potential conflict of interest.

## Footnotes

1 The photographs used in the current study were C-1-23, MF-1-26, MO-1-23, SW-2-30, EM-5-21, JJ-5-13, PE-3-21, WF-3-16 for “fearful” faces; C-2-18, MF-1-6, MO-1-4, SW-3-9, EM-4-7, JJ-4-7, PE-2-12, WF-2-12 for “happy” faces; C-2-3, MF-1-2, MO-1-5, SW-3-3, EM-2-4, JJ-3-4, PE-2-4, WF-2-5 for “neutral” faces.
